# Coherent Conformational Degrees of Freedom as a Structural Basis for Allosteric Communication

**DOI:** 10.1371/journal.pcbi.1002301

**Published:** 2011-12-08

**Authors:** Simon Mitternacht, Igor N. Berezovsky

**Affiliations:** 1 Computational Biology Unit/Uni Research, University of Bergen, Bergen, Norway; 2 Department of Informatics, University of Bergen, Bergen, Norway; Stanford University, United States of America

## Abstract

Conformational changes in allosteric regulation can to a large extent be described as motion along one or a few coherent degrees of freedom. The states involved are inherent to the protein, in the sense that they are visited by the protein also in the absence of effector ligands. Previously, we developed the measure binding leverage to find sites where ligand binding can shift the conformational equilibrium of a protein. Binding leverage is calculated for a set of motion vectors representing independent conformational degrees of freedom. In this paper, to analyze allosteric communication between binding sites, we introduce the concept of *leverage coupling*, based on the assumption that only pairs of sites that couple to the same conformational degrees of freedom can be allosterically connected. We demonstrate how leverage coupling can be used to analyze allosteric communication in a range of enzymes (regulated by both ligand binding and post-translational modifications) and huge molecular machines such as chaperones. Leverage coupling can be calculated for any protein structure to analyze both biological and latent catalytic and regulatory sites.

## Author Summary

What are the molecular mechanisms of allosteric communication in proteins? We base our analysis on the hypothesis that a folded protein has a number of conformational degrees of freedom, which describe fluctuations around the native conformation and switching from/to functional states. Transitions between the protein states involved in function and its regulation are based on coherent conformational degrees of freedom. Motion of one part of a protein along such a degree of freedom, implies a correlated motion in other parts of the protein. By determining which binding sites are simultaneously affected by the same motion we find sites that are allosterically coupled, i.e. where binding at one site can cause a change in ligand-affinity at another. Leverage coupling, the quantity introduced to measure this type of connection, reflects allosteric communication between different binding sites. We show how it can be used to understand allostery in enzymes of different sizes as well as in large protein complexes such as chaperones. Analysis of leverage coupling provides guidance in targeting native and latent regulatory sites.

## Introduction

The concept of allostery was originally formulated to describe cooperative ligand binding in oligomeric proteins. The first model of positive cooperativity in binding of oxygen to hemoglobin was proposed by Linus Pauling in 1935 [1], but the term allostery was coined in connection with the phenomenological MWC (Monod-Wyman-Changeux) and KNF (Koshland-Némethy-Filmer) models, developed in the 1960s [2], [3], [4]. Since then, there have been numerous studies of the mechanisms of allosteric regulation [5], [6], applying different experimental [7], [8] and computational approaches [9] to proteins as different as small single-domain enzymes, motor proteins [10] and chaperones [11], [12]. Although much progress has been made, the dichotomy between the original MWC and KNF models, or their modern counter parts, conformational selection and induced fit, dominates the discussion of allostery to this day [6]. The two models do however not describe mutually exclusive scenarios [13], [14], [15]: in both cases there is a shift in the population of different functional states upon effector binding. The main difference between the two is whether binding precedes conformational change or not [14]. Transition pathway analysis is primarily a matter of kinetics, whereas the shift in conformational equilibrium is one of thermodynamics: the conformational states involved determine which binding sites are allosterically connected, and their relative stability before and after binding determines the effect of regulation [6]. The major task therefore is to use this understanding to find structural determinants and molecular mechanisms of allosteric communication between distant binding sites [16].

Recently we developed the concept of *binding leverage* to measure the ability of a generic ligand, binding at different sites, to couple to conformational transitions, and thus its potential to have an allosteric effect [15]. We showed that in the majority of the studied cases, known allosteric and active sites had high binding leverage. We treated each site individually under the assumption that a site that has high binding leverage is connected to the global dynamics of the protein, without any specification of what other sites could be connected. Here we move on to investigate how allosteric communication takes place between specific pairs of sites. We introduce the concept of *leverage coupling*, which provides a quantitative characteristic of allosteric communication. We will also demonstrate how binding leverage and leverage coupling can be used to analyze allosteric communication mediated by metal binding and phosphorylation, as well as the function of three chaperones (GroEL-GroES, CCT and thermosome).

## Results

In this paper we will develop a molecular model of allosteric communication based on the concept of binding leverage (described in Methods). We recently showed that binding leverage could identify key binding sites, and also potentially latent allosteric sites, in a wide range of proteins [15]. Here we investigate how specific pairs of sites are allosterically connected via leverage coupling.

### Leverage coupling

To study site-site communication, we make the following assumption: *sites that have high binding leverage for the same motion are more likely to be allosterically coupled than sites that only have high binding leverage for motion along independent degrees of freedom.*

To represent a set of independent degrees of freedom we will use low frequency normal modes, which describe coherent motion involving the whole protein, and thus allow communication across large distances. We do not propose that protein dynamics is best described by global harmonic motion, but recognize the fact that the modes have repeatedly been shown to describe functional conformational change for proteins [17], [18]. We therefore use them as a set of basis vectors describing the allowed directions of motion around the folded state of a protein, and explore the possibility that movement along a given mode can have an independent functional relevance.

**Figure 1:**
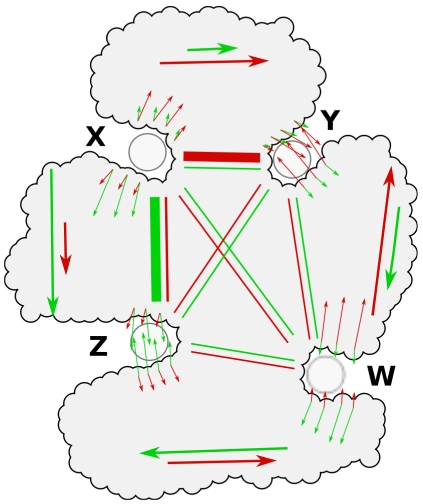
Illustration of the concept of sites communicating through leverage coupling.

We have illustrated the role of independent degrees of freedom in allostery for a toy protein in Figure 1. This protein has four binding sites W, X, Y and Z, and we have included two normal modes in the illustration, indicated by red and green arrows. The green mode causes closing of site Z and opening of site X, and only slight deformations of the other two sites. The red mode causes opening of site X and closing of site Y. Small red and green arrows indicate the deformation at each site for either mode. Site X and Y both have high binding leverage under the red mode and sites X and Z have high binding leverage under the green mode. This means that the pairs X and Y and Z and X are allosterically coupled, whereas the other pairs of sites are only weakly coupled (indicated by the thickness of the lines crossing the protein, connecting the corresponding sites). In practice, X could be a catalytic site, Z an activator site and Y an inhibitor site. There is only indirect competition between the effects of Z and Y, i.e. if an activator is present at Z the effect of an inhibitor at Y might be weaker, and vice versa. With other patterns of communication, there can of course also be cases where activator and inhibitor binding are mutually exclusive. Alternatively, if this protein was an oligomer, X, Z and Y could be identical sites with positive or negative binding cooperativity.

To quantify the strength of communication between two sites *P* and *Q*, as described in the previous paragraph, we introduce the *leverage coupling D_PQ_*. In the following, lower case roman indices (*i*, *j*) will number residues, lower case greek indices (*μ*, *ν*) normal modes, and upper case roman indices (*P*, *Q*) sets of residues, such as probe locations (see Methods) or biological binding sites. We denote the binding leverage of probe location *P* due to normal mode *μ* as *L_Pμ_* (see Methods). The symbol Δ*_iP_* is 1 if residue 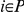, and 0 otherwise. The leverage *λ_iμ_* for a given residue and normal mode is then
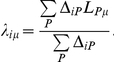



This calculation is done because our simulations generate a highly redundant set of probe locations, i.e. the denominator above can be large. Similarly, for an arbitrary set of residues *P*, we write
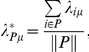
where the norm of *P* is the number of elements in the set. Next, we introduce the vector 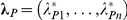, where *n* is the number of modes considered. The scalar product
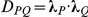
is large only if the sets *P* and *Q* have high leverage for the same normal modes. We will call the quantity *D_PQ_* the *leverage coupling* between the two sites. For example, for the two normal modes in Figure 1, *D_XY_* and *D_XZ_* are large, and *D_XW_*, *D_ZY_*, *D_ZW_* and *D_YW_* are small. Similarly, the matrix 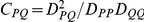 measures the normalized leverage coupling and has the range 0≤*C_PQ_*≤1. Since *D_PQ_* is based on normal mode vectors that represent infinitesimal motion, and depends on the size of the probe used in the calculation of *L_Pμ_*, the scale of leverage coupling values is arbitrary and unique to each protein. We therefore always compare the leverage coupling of specific sites to the average coupling between the residues not belonging to any sites, i.e. the background leverage coupling for a given structure.

The leverage coupling *D_PQ_* gives a measure of the strength of site-site coupling, but depends directly on the magnitude of conformational change at the different sites. In molecular machines like the chaperones, the conformational change at binding sites is small compared to the large-scale functional motions. Here, the measure *C_PQ_* can be used instead of *D_PQ_* to see how binding sites are correlated with different modes of functional motion. In this case we are interested in comparing the values between different sites and look for the most correlated pairs of sites for a given protein. The range of color bars in all figures containing *C_PQ_* matrices is from 0 to 1, which reflects the span of *C_PQ_* values. Finally, the special case where one of the sets only has one residue can be used to see how one site couples to the rest of the protein. We will denote this variant *D_Pi_*, where *P* is the studied site and the index *i* runs over all residues.

### Analysis of allosteric enzymes

We study 15 enzymes regulated by ligand binding, 14 of which were studied in our papers on binding leverage [15] and local closeness [19]. The addition is the 20-meric enzyme GTP cyclohydrolase I (GTPCHI), which is both activated and inhibited allosterically by different substances [20], [21]. These 15 enzymes are supplemented by 5 additional proteins, to generalize the analysis to other types of regulation and non-enzymes: Glycogen phosphorylase (GP) is allosterically regulated by both phosphorylation and ligand-binding [22]. The serine-protease thrombin is allosterically regulated by sodium binding [23]. The type I (GroEL-GroES) and type II (CCT, thermosome) chaperones are molecular machines regulated by ATP binding and hydrolysis [24]. The simulation parameters for the proteins discussed in the main text are summarized in Table 1. The binding leverage was calculated using the ten lowest frequency normal modes [15]. The analysis of all the other proteins in this paper is based on the calculations described in the above paper.

**Table 1:** Simulation parameters and results.

Protein	PDB codes	# residues	Probe size	# sim	MC steps
AdK	4ake, 1ake	214	8	500	50 000
PFK	3pfk, 4pfk, 6pfk	1276	4	4 000	200 000
GFRP-GTPCHI	1wpl, 1is7, 1is8	2780	4	20 000	600 000
GTPCHI	1wpl (chains A–J)	1940	4	10 000	400 000
GP	1gpa, 1gpy, 1a8i	1594	4	8 000	400 000
Thrombin	1sgi, 1sg8	277	2	1 000	100 000
GroEL-GroES	1sx4	8014	4	30 000	1 500 000
CCT	3p9d	8370	4	30 000	1 500 000
Thermosome	1a6d, 1a6e	8040	4	30 000	1 500 000

“# sim” refers to the number of simulations performed, i.e. the number of probe locations generated. “Probe size” refers to the number of atoms in the probe.

**Figure 2:**
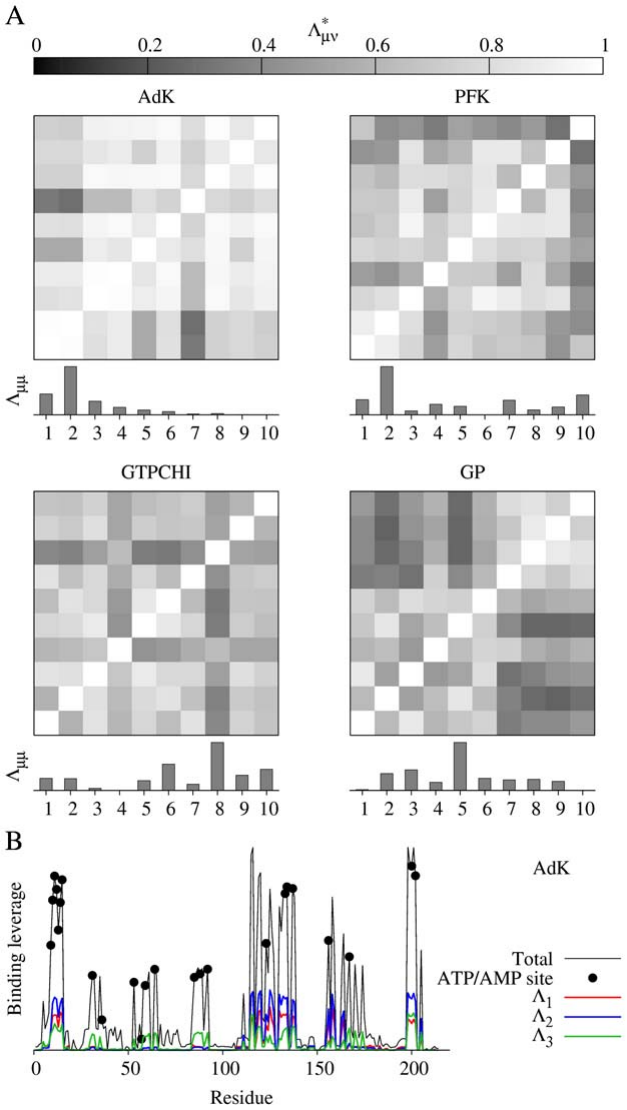
Leverage profile properties. (A) The matrix 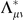 measuring similarity between leverage profiles for different normal modes *μ* and *ν*, for 4 of the proteins studied. The magnitude of the leverage profiles 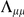 are also plotted to indicate which are the most important modes. (B) The total leverage profile and the three most important individual leverage profiles for AdK (**Λ**_1_, **Λ**_2_, and **Λ**_3_). The average total binding leverage for the residues at the active site are indicated by black circles.

To begin with, we will briefly try to give the reader some intuition of what the leverage profiles can look like and how they relate to each other. The leverage profile similarity 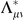 (defined in Methods) for the 10 lowest frequency normal modes, excluding the trivial first six modes, is plotted in Figure 2A for four different proteins. A value of 1 indicates that the two corresponding modes affect the exact same sites, and 0 that there is no overlap. Also included in the same panel is the importance of each of these normal modes, Λ*_μμ_* (see methods). Like for leverage coupling, the scale of leverage profiles is arbitrary and only relative values are relevant. For adenylate kinase (AdK), the most significant leverage profiles correspond to modes 1, 2 and 3. Of these profiles, **Λ**_1_ and **Λ**_2_ are very similar. Figure 2B shows that these two leverage profiles peak at the same position, whereas the third is spread over more residues. That the leverage profiles are similar means that binding leverage is high for the same sites under the corresponding normal modes, even though these modes are orthogonal. Also included in the figure is the total binding leverage along the sequence, which is the sum of *λ_iμ_* over all modes *μ*. Almost all active site residues (involved in ATP and AMP binding) are located at peaks in the total binding leverage.

Having verified that different sites have their highest binding leverage for different normal modes, we move on to the analysis of leverage coupling. Supplementary Figure S1 contains plots of the leverage coupling matrix *D_PQ_* for the proteins not discussed in detail in the main text. The figure illustrates that, with the exceptions of ATCase and PTP1B, which we showed were difficult to analyze with binding leverage [15], there is generally a stronger coupling between at least some of the allosteric and active sites (including homotropic communication) than between these sites and the rest of protein. One can also see that some sites are more strongly coupled than others are. We will however not analyze these proteins in detail; instead, we will focus on a couple of noteworthy cases.

**Figure 3:**
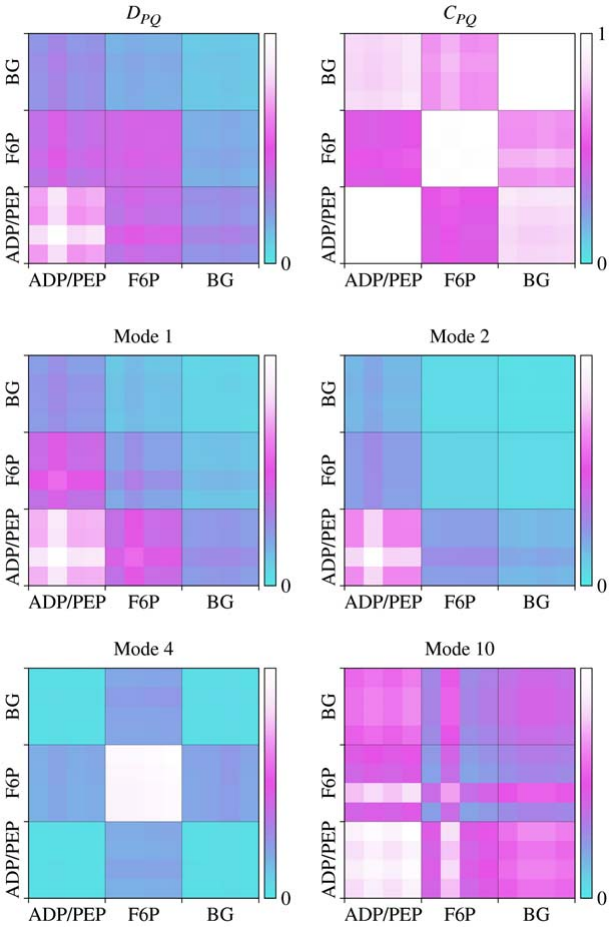
*D_PQ_* and *C_PQ_* matrices for PFK. The single mode matrices for PFK were calculated like *D_PQ_* but only using one normal mode. The color runs from 0 (cyan) to the maximal measured value (white) for *D_PQ_* and from 0 to 1 for *C_PQ_*.

The tetrameric enzyme phosphofructokinase (PFK) in *Bacillus stearothermophilus* has one regulatory site where it is activated by ADP binding and inhibited by phosphoenolpyruvate (PEP) binding. The individual low frequency normal modes for this protein are less similar to each other than for AdK and there are also more modes that contribute significantly to binding leverage (Figure 2A). In Figure 3 we display the leverage coupling *D_PQ_* for the four effector sites (*P* = 1–4, ADP/PEP), the four active sites (*P* = 5–8, F6P) and the remaining residues of the four chains (*P* = 9–12, BG). As indicated by the color bars, the figure displays values from 0 to the maximal value of leverage coupling measured, in each matrix. Interactions between the effector sites dominate the matrix, and interactions between effectors and active sites are also strong, whereas interactions between the four active sites are weak. The latter indicates that there could be cooperative binding of effector but not of substrate. Experiments have shown that substrate binding is only cooperative in the presence of PEP [25].

The normalized leverage coupling *C_PQ_* is high if the sites *P* and *Q* have their peaks in binding leverage for the same modes. The *C_PQ_* matrix in Figure 3 for PFK indicates that different sets of modes affect the effector and active sites – the correlations are strong within the two groups of sites, but weaker between them. To demonstrate the validity of this interpretation we also included the *D_PQ_*-matrices for four of the individual modes. The modes were chosen from the dominating ones in Figure 2A. Modes 1 and 2 primarily affect the effector sites. Mode 1 also involves some connections between effectors and substrate. Mode 4 essentially only affects the active sites, and is probably responsible for any (weak) substrate binding cooperativity. Mode 10 provides relatively strong connections between the active site and the allosteric site, and Figure 2A shows that this is the second most important mode.

**Figure 4:**
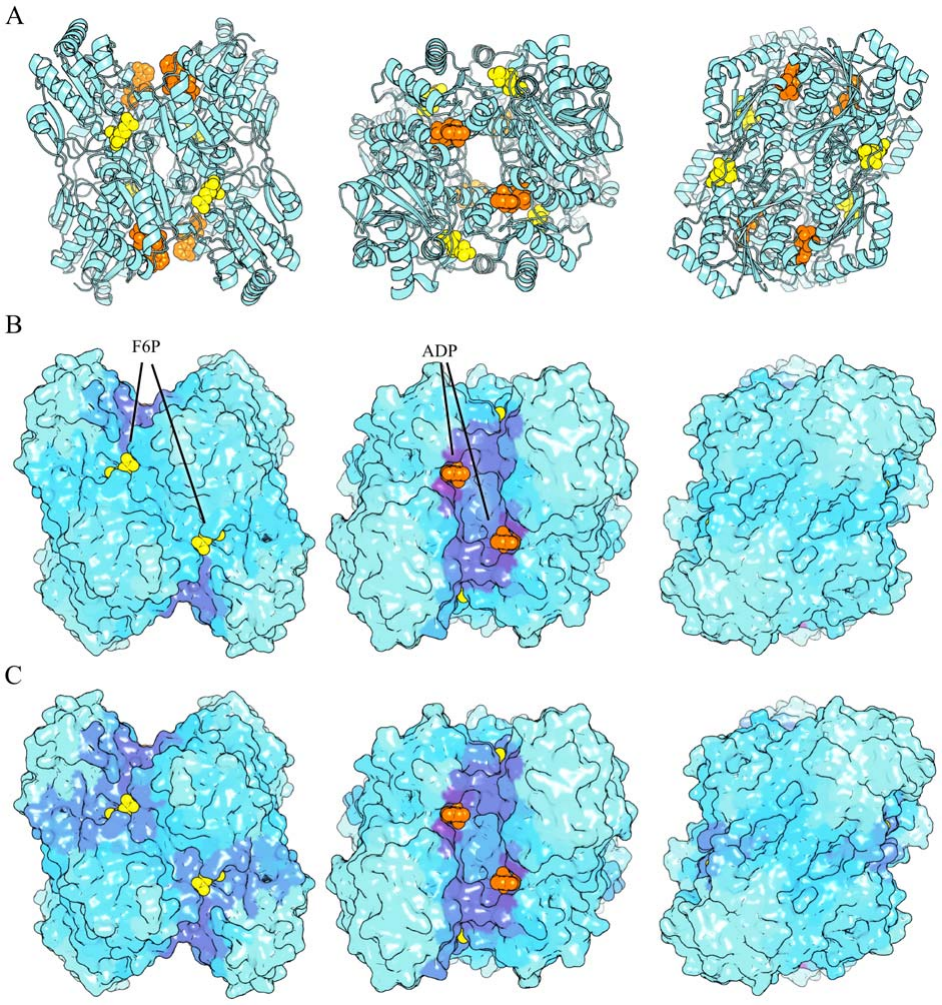
Phosphofructokinase (PFK). All 3D structures in this paper were drawn with PyMol. (A) Structure of PFK (PDB entry 3pfk). The effector ADP is drawn with orange spheres, and the substrate F6P with yellow spheres, ligand coordinates were taken from PBD entry 4pfk. (B) Leverage coupling *D_Pi_* between ADP site of one chain (lower right ADP) and the rest of the protein. The surface is colored in a gradient from cyan to magenta where cyan represents the lowest measured value of *D_Pi_* and magenta the highest value. (C) Same as (B) but for one of the F6P sites (lower right one).

To illustrate the communication between sites we color the surface of the protein by the leverage coupling between one site and each residue of the protein, *D_Pi_* (see Methods) in Figure 4, the raw data can be found in Figure S2. The coloring in this figure, and in similar ones below, uses cyan for *D_Pi_* = 0, and magenta for the maximal value of *D_Pi_* over all residues *i* for a given site *P*, i.e. the coloring gives the pattern of communication for a given site, but no indication of coupling strength compared to other sites *P*. The studied effector site in PFK communicates most strongly with the other effector sites (Figure 4B), whereas the active site is connected with the other active sites, as well as the allosteric site (Figure 4C). This apparent asymmetry comes from the fact that the interaction between effector sites is stronger than between anything else, but the connection between the active site and the effector site has approximately the same strength as the connections between active sites. Noteworthy is also the fact that neither site has any strong connections to sites other than the functional ones.

**Figure 5:**
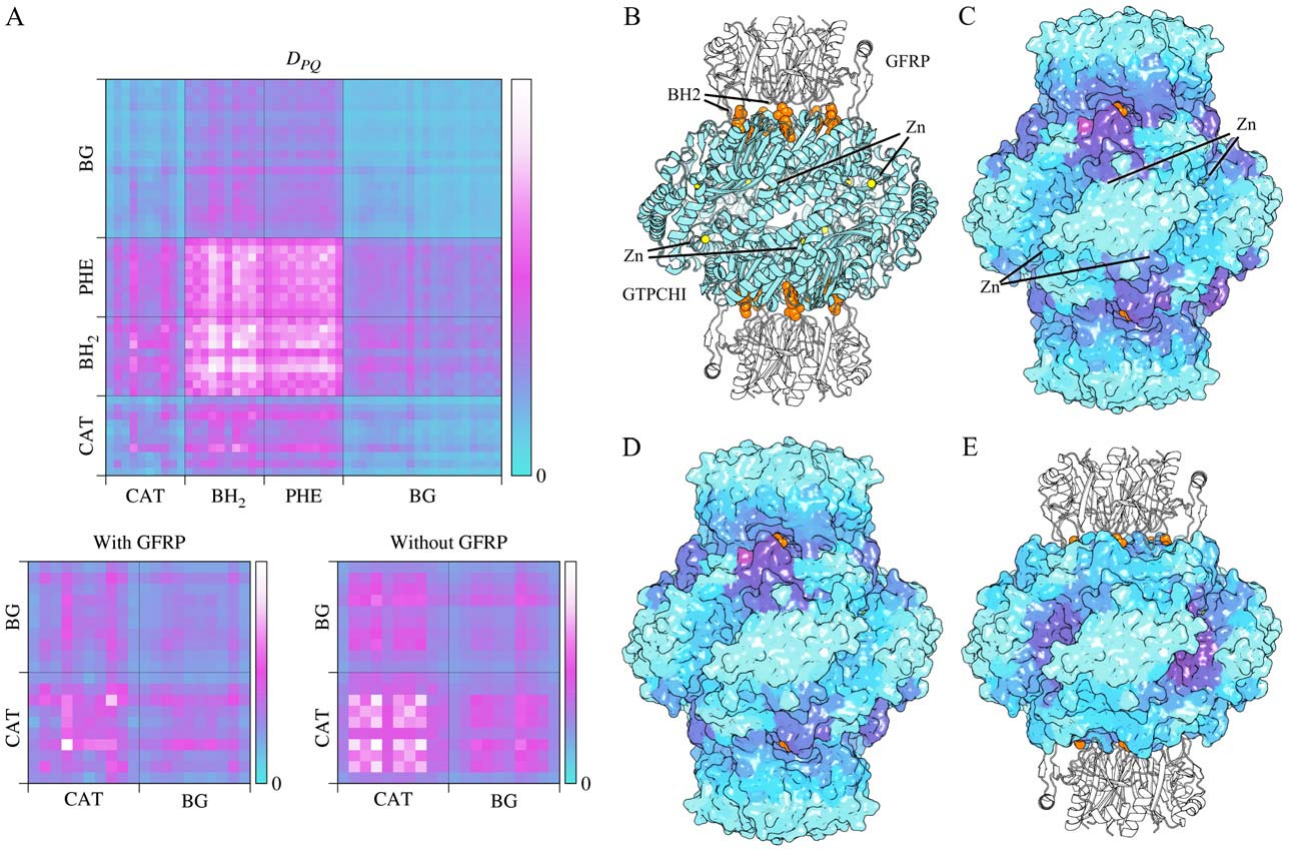
GTP cyclohydrolase I (GTPCHI) with feedback regulatory protein (GFRP). (A) Top: the matrix *D_PQ_* for the whole protein. Bottom left: selected sections of the top matrix. Bottom right: same section as left panel, but calculated for structure without GFRP. (B) Structure (1wpl). The GTPCHI decamer is drawn in cyan, and the two GFRP pentamers in white. The inhibitor BH_2_ is drawn with orange spheres and the Zn at the catalytic site in yellow. (C) Communication *D_Pi_* between one BH_2_-site and the rest of the protein. The color scheme is the same as in Figure 4. (D) Communication between one of the active sites and the rest of the protein. (E) Same as (D) but normal modes and docking calculations were done without GFRP.

GTPCHI catalyzes the first step in the production of tetrahydrobiopterin (BH_4_) from GTP. It has positive cooperativity with respect to GTP binding. Allosteric regulation depends on the presence of the GTPCHI feedback regulatory protein (GFRP). In combination with phenylalanine, GFRP reduces the cooperativity of GTP binding, increasing the activity at low GTP concentrations [20]. The GFRP-GTPCHI complex can also be inhibited by BH_4_ [21]. Both BH_4_ and phenylalanine bind at similar locations at the GTPCHI-GFRP interface. The architecture of the GFRP-GPTCHI complex is illustrated in Figure 5B. GTPCHI is a homodecamer arranged in two pentameric rings, and the regulatory GFRP pentamers bind one to each ring.

We analyze three sites in the GFRP-GTPCHI complex, the BH_4_ site (BH_2_ in the crystal structure), the phenylalanine site (PHE) and the catalytic site (CAT). We define the catalytic site as all residues interacting with the catalytic Zn, and also His-134 and His-201 as defined in the catalytic site atlas [26]. The two allosteric sites have overlapping locations at the GFRP-GTPCHI interface and therefore have large mutual leverage coupling, as can been seen in Figure 5A, but both also couple strongly to the active site. The coupling between catalytic sites is not very strong in this complex, which is consistent with the fact that GFRP and Phe reduce cooperativity. To test the role of GFRP in modifying cooperativity in terms of binding leverage we removed GFRP from the structure and redid the calculations. The bottom two panels of Figure 5A show the coupling between the 10 different catalytic sites with and without GFRP. The effect is not very strong, but it is clear that the GTPCHI catalytic sites in the structure without GFRP are more strongly coupled compared to the background, than in the structure with GFRP.

The connections *D_Pi_* between one of the allosteric BH_4_-sites and the rest of the protein are illustrated in Figure 5C (raw data in Figure S3). Similarly, the coupling to one of the active sites, with and without GFRP present, is shown in Figure 5D and E. In the GFRP-GTPCHI complex the regulatory sites and their surrounding residues have the strongest leverage coupling, as was also seen for the site-site coupling matrix *D_PQ_*. This figure however clearly illustrates that communication with the “background” only involves the surroundings of the effector binding sites, and does not involve any other distinct sites.

### Allosteric regulation involving metal binding and phosphorylation

The concepts of binding leverage and leverage coupling can be generalized to study other forms of allosteric communication. Therefore, we consider cases of regulation involving metal binding and phosphorylation.

**Figure 6:**
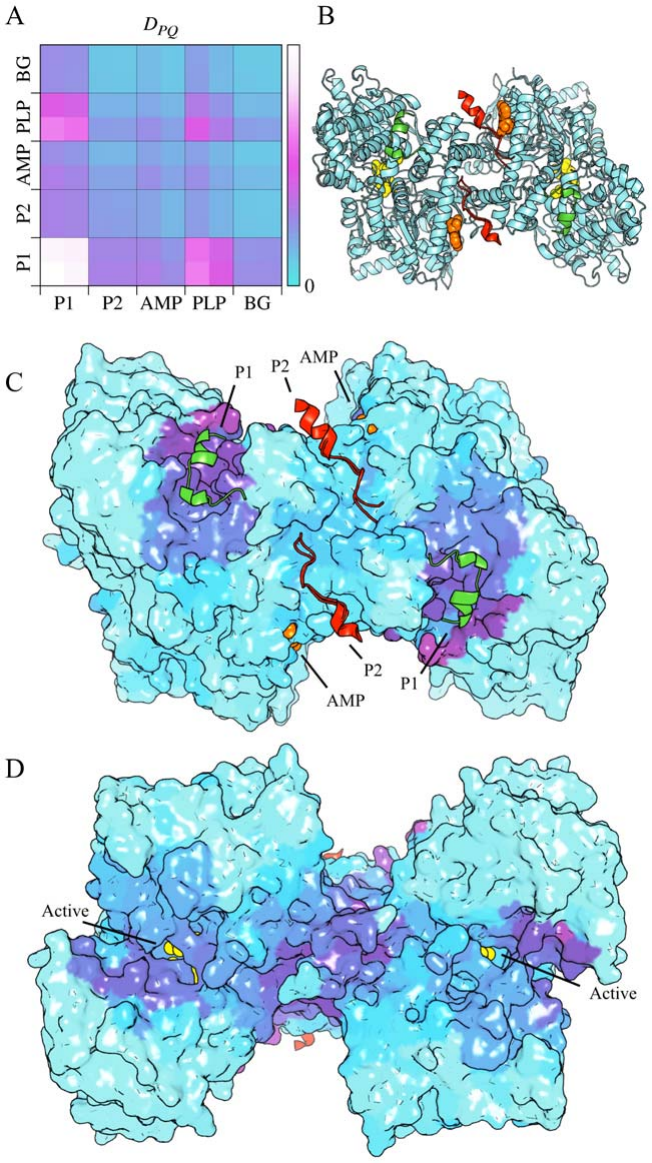
Glycogen phosphorylase (GP). (A) *D_PQ_* matrix with AMP and PLP sites, plus the locations for the segment 1–20 in GPb (P1) and GPa (P2). (B) Structure of GPa (1gpa). The segment 1–20 that moves upon phosphorylation of Ser14 is green in the GPb form and red in the GPa form. The slightly hidden coenzyme PLP and the substrate GLS are drawn as yellow spheres. (C) and (D) Two views of the coupling *D_Pi_* between active site and the rest of the protein. The color scheme for *D_Pi_* is the same as in Figure 4.

We study glycogen phosphorylase (GP) as a case of allosteric regulation via covalent modification. Glycogen phosphorylase has two main conformations: the inactive dimeric T state and the active tetrameric R state [22], [27]. In addition, it has two forms, GPa and GPb, where the former is phosphorylated at Ser14. Crystal structures are available for both R and T state forms of GPa and GPb, but the R state is favored for unliganded GPa, and the T state for unliganded GPb. Both GPa and GPb are heterotropically activated by AMP, and inhibited by ATP and other metabolites. Upon phosphorylation, residues 1–20 become more ordered and move to a new position, 30 Å or so away, as can be seen in Figure 6B.

In our calculations we use crystal structures of rabbit muscle GP. PDB entry 1gpa, representing unliganded GPa, is used for normal mode calculations; in addition we use T state GPb (1a8i) and AMP-activated R state GPb (3e3n) to define different binding sites. To be able to analyze phosphorylation using binding leverage, we treat residues 10–20 as a peptide ligand binding at two different sites, P1 (T state GPb) and P2 (R state GPa), and calculate the normal modes without the 20 first residues. Figure 6A shows *D_PQ_* for P1 and P2, and also the active (PLP) and allosteric sites (AMP). It is clear that the connections are strongest between P1 and the PLP site. There is an unexpectedly weak interaction between the AMP and PLP site. Since P1 seems more important than P2 we hypothesize that release of residues 1–20 upon phosphorylation from P1 is more important for allostery than binding to P2. The role of P1 is however somewhat uncertain given that residues 1–20 are relatively disordered in GPb. The connections are more or less symmetric between chains indicating that phosphorylation of one chain can trigger a global conformational change. To illustrate the connections between the active site and the rest of the protein we have drawn *D_Pi_* for the active site in Figure 6C and D (raw data in Figure S4). This figure clearly shows strong connections between the active sites themselves and with P1, but also towards one side of the dimer interface, opposite to P2, which could contain latent allosteric sites.

We also analyzed yeast glycogen phosphorylase (yGP), which is structurally very similar to rabbit muscle GP, but differently regulated. The N-terminal strand in yGP is 40 residues longer than in rabbit muscle GP. In the GPb form the strand binds to the active site instead of P1, and in the GPa form it folds at the dimer interface, at a position similar to P2 above [28]. The differences in regulatory mechanism between these two proteins are thus primarily due to the differing length of the N-terminal strand. This strand is excluded in our calculations and we therefore do not expect any qualitative differences between the two variants. We analyzed yGP using the same parameters as above, based on PDB entry 1ygp, having removed all residues before position 22 (using the 1ygp numbering). We found that the leverage coupling between the active site and the rest of the protein is essentially identical to that of rabbit muscle GP, indicating that P1 is a latent allosteric site in yGP (data now shown).

As an example of metal binding-induced allostery we study the serine protease thrombin which is allosterically regulated by sodium binding [23]. It is also controlled by two other allosteric sites: exosite I (EX1) interacts with several different protein partners, and exosite II (EX2) interacts with several polyanionic substrates [23]. We divide the active site into three groups, the catalytic triad (CAT) and two of the substrate recognition pockets P2 and P4. The leverage coupling of this protein is shown in Supplementary Figure S5. The binding leverage of the sodium site is very low, and coupling to other sites weak. The sodium-induced conformational change primarily involves side-chain rearrangements, which are not modeled by our procedure. The concept of binding leverage could be expanded to include side-chains at a significant computational cost. Single side-chain rearrangements are however not expected to be modeled by low frequency normal modes, which means that a more refined description of motions would probably also be required to model the sodium regulation.

### Analysis of chaperones

Above, we analyzed a set of enzymes, some of them very large with up to 3 000 residues (GTPCHI-GFRP, ATCase and GDH), and found that leverage coupling gives an understanding of allosteric communication in these enzymes. To push the envelope even further we will now move to the chaperonins, molecular machines with about 8 000 residues. These large molecules are quite challenging to study, the main bottleneck in our analysis being the time required to generate the very large number of probe locations needed, and the calculations took roughly 30–40 CPU hours for each chaperone on a modern desktop PC.

Chaperonins represent a different type of allostery compared to the homo- and heterotropic regulation seen in enzymes. These molecular machines cycle through a set of conformations to provide a protected chamber for protein folding. ATP binding and hydrolysis cause large conformational changes to facilitate substrate capture, folding and release [24]. We will analyze and compare the bacterial group I chaperonin (GroEL-GroES) and eukaryotic and archaeal group II chaperonins (CCT, Thermosome) to investigate differences in regulatory mechanisms.

The concepts developed in this paper were designed to analyze coupling between distinct ligand binding sites in enzymes, but, given a regulatory site, we can detect which parts of the protein are likely to have conformational change coupled to binding at that site. When a domain is deformed, the domain itself does not have high binding leverage, but many of the domain's hypothetical binding sites do. In this context binding leverage is therefore rather a measure of the degree of deformation of a section of the protein. By computing the leverage coupling *D_PQ_* for a site *P* and a domain *Q*, we can see how binding at the site *P* couples to conformational change in domain *Q*, making it possible to analyze allosteric communication in molecular machines such as chaperones.

**Figure 7:**
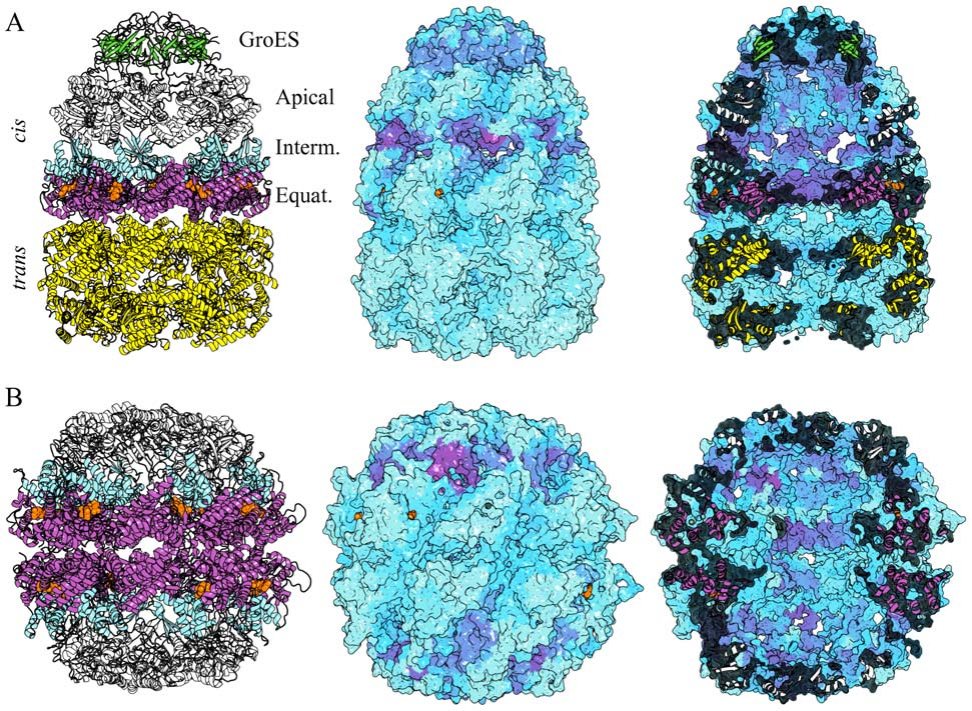
Chaperones GroEL-GroES and CCT. (A) Left: Structure of GroEL-GroES colored by the different domains (PDB entry 1sx4). Middle and right: surface and cross-section of GroEL-GroES displaying coupling between one ATP site and the rest of the protein *D_Pi_*. ADP molecules are displayed as orange spheres throughout. The ATP site used for the calculation is the second one from the left in the present view of the *cis* ring (B) Left: Structure of CCT chaperone (PDB entry 3p9d) with subdomains and ligands colored analogously to GroEL-GroES. Middle and right: *D_Pi_* for the second ATP site from the left in the upper ring. The color scheme for *D_Pi_* is the same as in Figure 4.

The GroEL-GroES chaperone consists of two heptameric rings (GroEL) and a heptameric lid (GroES) attached to one of the GroEL rings (see Figure 7). The ring closest to GroES is called the *cis*-ring and the other the *trans*-ring. Each GroEL ring provides a folding chamber. The functional cycle roughly goes through the following steps [24], [29]: After substrate has bound to one of the open GroEL rings, ATP binds cooperatively to the GroEL ring [30] and increases affinity for GroES [31]. GroES binding causes a large conformational change increasing the volume of the *cis* folding chamber and changing it from hydrophobic to hydrophilic [24], allowing folding to take place [32]. ATP hydrolysis weakens the affinity for GroES and when substrate and ATP have bound to the *trans* ring GroES and substrate are released from the *cis* subunit. In addition to intra-ring communication, there is also inter-ring signaling, which (i) adjusts the *trans* ring to accept substrate after *cis* ATP hydrolysis; (ii) leads to the ejection of *cis* substrate as a result of *trans* ATP binding [33]; (iii) accelerates the ejection of *cis* substrate by simultaneous binding of ATP and polypeptide to the open *trans* ring [34]. According to cryo-EM analysis, the equatorial domains play a key role in the inter-ring signaling [35]. Here, we will study the allosteric communication between the *cis* ATP sites and the rest of the protein.

**Figure 8:**
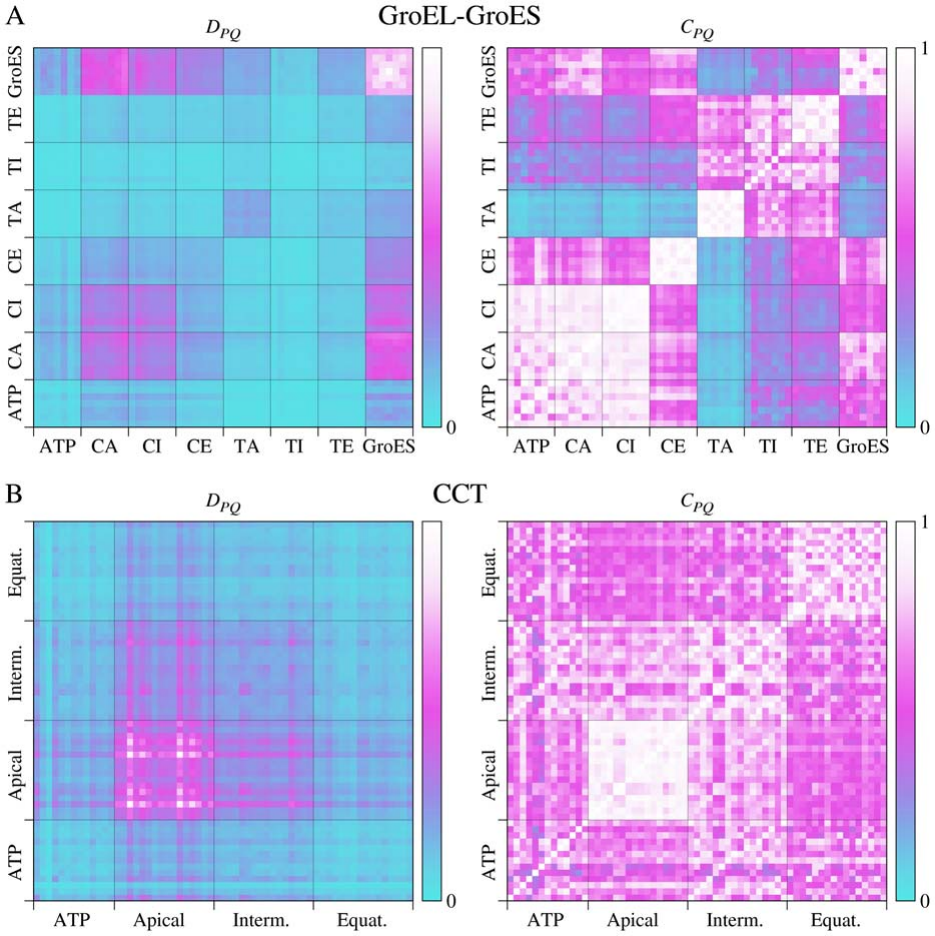
Site-site communication in chaperons. (A) Matrices *D_PQ_* and *C_PQ_* for GroEL-GroES complex. Equatorial, intermediate and apical domains are marked CE, CI, CA and TE, TI, TA for the *cis* and *trans* rings respectively. (B) Similar to (A) but for CCT. All 16 subunits have been divided into three subdomains, but there were only 13 ATP analogs bound to the crystal structure.

Conformational changes in GroEL involve the equatorial, intermediate and apical subdomains (see Figure 7). ATP binds to the equatorial domain and GroES to the apical domain. ATP binding controls the expansion of the folding chamber which takes place when the intermediate domain swings away from the equatorial domain. The apical domain follows the intermediate domain in this motion, largely as a rigid body. ATP hydrolysis mainly induces an increased flexibility of the intermediate and apical domains [29], which probably explains the looser attachment of GroES to GroEL-ADP_7_ than to GroEL-ATP_7_. ATP binding and hydrolysis is positively cooperative within each ring and negatively cooperative between the rings, providing tight ATP binding to only one ring at a time [36]. Figure 8A shows the leverage coupling *D_PQ_* and the normalized *C_PQ_*, for the ATP sites, the three subdomains of the *cis* ring, the *trans* ring and GroES. The strongest connections are between the chains of GroES. Second in strength are the connections between the apical and intermediate domains and GroES, and between the apical and intermediate domains themselves. The ATP site is also only weakly connected to the protein, a result of the fact that the equatorial domain and the ATP site undergo much smaller conformational change than the other two domains. The normalized leverage coupling *C_PQ_* however shows that the ATP site is more correlated with the apical and intermediate domains than with the equatorial domain to which it belongs. Correspondingly, there are strong correlations within the *trans* ring, where the magnitude of leverage coupling is much lower. The high degree of symmetry of the subsquare of the *C_PQ_* matrix describing interactions between the ATP site and the intermediate domain, and partly also the apical domain, is consistent with the positive cooperativity observed for ATP binding within one ring. Finally, there is a weaker correlation between the *trans* equatorial and intermediate domains, and the *cis* ring, particularly between the equatorial domains of either ring. These connections could be involved in the negative cooperativity between the two rings.

We also analyzed the coupling *D_Pi_* for one of the ATP sites, two views of this measure are provided in Figure 7A (raw data in Figure S7). The coloring indicates that the inside of the *cis* cavity, the GroEL-GroES interface and the interface between apical and intermediate domains are most strongly communicating with the *cis* ATP site. There is hardly any connection to the *trans* ring. These findings should be related to the fact that the main function of ATP is to regulate the *cis* cavity and the interactions with GroES, and also to the positive cooperativity of ATP binding.

The human chaperone CCT has a similar function to GroEL, but does not utilize an analog to GroES. It consists of octameric rings, with similar but non-identical chains, instead of heptameric ones. It is also regulated by ATP binding and hydrolysis with steps similar to those of GroEL [24]. ATP binding is not cooperative, regulation has been described as sequential rather than concerted [37]. This is also reflected in the fact that only a fraction of the 16 ATP pockets were populated in crystal structures (13 in the one we use). The leverage-coupling matrix in Figure 8B shows that some of the apical domains are strongly coupled to each other, but coupling between intermediate domains is weaker. The normalized leverage coupling matrix in the same figure, *C_PQ_*, indicates that ATP has a weaker correlation with the apical and intermediate domains in CCT than it does in GroEL. In this plot the chains are ordered alphabetically, i.e. the first eight elements along either axis for each domain (apical, intermediate, equatorial) belong to the same ring, and the last eight to the other. This means that for CCT, interactions between the rings are as strong as within them, which is clearly different from what we saw for GroEL where the *trans* ring was only weakly connected to the rest of the protein. On the other hand, in CCT there is a greater asymmetry in the allosteric connections within one ring than in GroEL-GroES, in particular between the ATP site and the intermediate domain. This asymmetry is seen from the anisotropy of the different subsquares of the *C_PQ_* matrix, and is consistent with the sequential regulation of this chaperone [37].

Figure 7B shows the leverage coupling *D_Pi_* for one of the ATP sites of CCT (raw data in Figure S7). As for GroEL-GroES (Figure 7A), the ATP site is more strongly connected to the inside of the cavity than the outside, but in this case the pattern is relatively symmetric between the rings. The strongest deviation from symmetry, and also the strongest visible leverage coupling, is to a nearby interface between intermediate and apical domains (magenta area in the middle panel of Figure 7B).

The archaeal thermosome is homologous to CCT, but has a higher degree of symmetry than CCT [38]. The results of the analysis of this protein can be found in Figures S6 and S7. The leverage coupling *D_PQ_* and the normalized *C_PQ_* in Figure S6A shows a pattern similar to CCT; the communication between apical domains is strong, and ATP is more strongly connected to the intermediate domain than the equatorial domain. The thermosome however displays a higher degree of symmetry (as indicated by the uniformity of subsquares in the matrices). The *D_Pi_* surfaces for one of the ATP sites in Figure S6B also shows a higher degree of symmetry than for CCT; in particular, the coupling to the neighborhood of the studied site is not stronger than to the rest of the protein. The difference in the symmetry of *D_Pi_* is especially clear when comparing the two corresponding curves in Figure S7. Symmetry is usually associated with positive cooperativity: the difference in symmetry between CCT and the thermosome might therefore reflect a difference in cooperativity, within the rings.

Comparing to previous computational works [11], [12], [29], [39], [40], we analyze allosteric communication between subunits in complete structures of both group I and group II chaperonins. It allows us to detect symmetry in the interactions between subunits of the *cis* ring of GroEL-GroES and its absence in CCT. We show that leverage coupling helps to understand positive cooperativity in the *cis* ring and negative cooperativity in the inter-ring communication in GroEL-Gro-ES, non-cooperative mechanism in human CCT, as well as positive intra-ring cooperativity in archaeal thermosome.

## Discussion

Despite the almost half-century long studies of allostery, the majority of the works represents analysis of individual proteins (or groups of homologs) and mechanisms of allostery characteristic for individual structures. In this work, we sought a structural characteristic that can be used to understand allosteric communication in proteins of different types and sizes, from small single-domain proteins to large multi-chain oligomers and chaperones. We resort here to the thermodynamic aspect of allosteric regulation, where the conformational equilibrium between different structural states and their relative stability determine allosteric communication between sites and effect of regulation. We developed the concept of leverage coupling based on the idea that long-range communication between allosteric sites can be mediated by coherent motion along independent conformational degrees of freedom. We have studied the allosteric regulation of a number of proteins controlled by ligand binding, phosphorylation, or metal binding. The analysis has provided new insight into the allosteric mechanisms involved. Two approaches to the problem have been applied, first an analysis of known biological sites, to see how they are connected to each other, and how coupling between them compares to the background. Second we have selected specific sites and analyzed how these are coupled to the rest of the protein, thus being able to identify important functional regions of the protein, that are communicating with these specific sites, and in some cases see how different sites are coupled to different parts of the protein.

We began our analysis by showing that leverage coupling largely captures the important connections in a number of enzymes, and exemplified this for phosphofructokinase (PFK) and GTP cyclohydrolase I (GTPCHI). We also showed that the role of GFRP in regulating homotropic cooperativity in GTPCHI was described well by leverage coupling. In the case study of allostery by phosphorylation in glycogen phosphorylase, we found indications that the active sites had high leverage coupling with the site where the unphosphorylated N-terminal segment binds (in a low temperature crystal structure), and hypothesize that the release of this segment upon phosphorylation causes the functional regulation. Allosteric regulation by metal binding in thrombin can however not be explained by leverage coupling, at least not in the coarse-grained version employed here. Finally, we have demonstrated that leverage coupling can be used to analyze allosteric communication in three different chaperones, and captures the differences in cooperativity between CCT and GroEL-GroES. We were able to describe allosteric communication between structural subunits providing positive cooperativity within each ring and negative cooperativity between the rings via inter-ring communication.

The concept of allosteric communication mediated by collective degrees of freedom, as presented here, is based on our understanding of the physical principles determining protein dynamics. Using normal modes and coarse-grained docking simulations is a crude approximation of these principles – a complete description of the processes involved requires a statistical mechanics analysis based on a reliable energy function and proper conformational sampling. However, our analysis is successful in identifying communicating pairs of sites in the majority of the studied proteins, supporting our assumption that allosteric regulation relies on coherent conformational changes of oligomeric proteins and their domains. We have furthermore demonstrated that different regulatory sites have different patterns of communication (see for instance the difference between active and allosteric sites in PFK), which are determined by motion along independent structural degrees of freedom, in our case different normal modes. This finding gives strong support to the idea that the ability of particular sites to couple to certain modes of motion, and not others, as illustrated in Figure 1, can provide directed and differential allosteric communication and regulation. We have thus moved beyond the framework defined by the classical KNF and MWC models, both in that we propose a molecular mechanism for connecting different sites, and in that we are able to predict and identify many functional sites. Using normal modes to represent independent conformational degrees of freedom, we find that these motions can be used not only to describe the allosteric transition geometrically – as many have done before – but also to explain allosteric connections between different binding sites and to identify latent allosteric sites. Novel allosteric connections predicted by leverage coupling can be used as targets in experimental inhibitor/activator design.

## Methods

The calculation of binding leverage involves two main steps, generation of possible ligand conformations through coarse-grained Monte Carlo simulations, and analysis of the generated binding sites with respect to motions deduced from one or more crystal structures [15]. Probe conformations in which the probe is highly stressed, under a given protein motion, have high binding leverage. Binding leverage models allostery based on the assumption that binding to sites where ligand-protein interactions are connected to important degrees of freedom can affect the conformational equilibrium. We used binding leverage to rank probe locations (defined below) and found that high-ranking probe locations matched active and allosteric sites in a wide range of proteins. Here, we will give a brief overview of the procedure, which was described in detail previously [15].

Ligand binding is simulated with a completely fixed Cα-representation of the protein chain and a freely moving probe ligand in the form of a peptide with one or more Cα-atoms. The probe and protein interact via a square well potential which is attractive for Cα-Cα distances between 5.5 and 8 Å. Distances shorter than 4.5 Å are forbidden. Potential binding sites, called probe locations, are generated by running a number of short docking simulations. A *probe location* is defined as the residues interacting with the probe at the end of a given simulation. Binding leverage measures the ability of a probe ligand to resist a given motion, for example that of a normal mode. A spring is placed between all residue pairs in a probe location whose interconnecting lines pass through the ligand. The binding leverage of a probe location is then calculated as the total change in spring potential energy *U* due to a given motion, i.e.
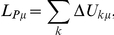
where summation is over all springs, and the additional index *μ* numbers the motion vectors used, i.e. one leverage is calculated for each vector. If more than one motion is considered the binding leverage can be summed to a total binding leverage for the probe location.

Cα normal modes were calculated using MMTK with default parameters for all cases [41]. For the large proteins GTPCHI, GroEL-GroES, CCT and the thermosome we used the Fourier-basis approximation [42], in all other cases vibrational modes are used.

The binding leverage 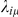 of residues *i* under mode *μ* (defined in the main text) can be grouped into leverage profiles 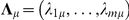, where *m* is the number of residues. We write the scalar product between two profiles as
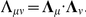



The magnitude Λ*_μμ_* of a leverage profile indicates the importance of the corresponding normal mode in the total binding leverage, and the normalized scalar product
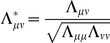
is close to one when the corresponding modes involve the same binding sites, and close to zero when the overlap is small.

## Supporting Information

Figure S1 Leverage coupling *D_PQ_* for the proteins not described in the main text. The data is based on our work on binding leverage, where simulation parameters for the respective proteins can be found [15]. The label “BG” corresponds to the background, which is an average leverage coupling calculated over all residues not belonging to any site. The other abbreviations designate allosteric and functional sites, using the three letter codes found in the PDB-files for ligands binding at those sites. Anthranilate synthase (1i7s): BEZ/PYR - substrate; ILG - substrate; TRP - inhibitor. ATCase (3d7s): ATP and CTP - effectors; PAL - substrate. DAHPS (1gg1): PGA - substrate; PHE - inhibitor. G6PD (1cd5): 16G - activator; AGP - substrate. GDH (1nr7): ADP - activator; GLU - substrate; GTP - inhibitor; NDP - coenzyme. NADME (1gz3): ATP – active site ligand; FUM - activator. PGDH (1yba): AKG - substrate; NAD - coenzyme; SER - inhibitor. PTP1B (2hnp): 892 - inhibitor; BPM - substrate. SSUPRT (1xtt): CTP - inhibitor; U5P - substrate. Threonine synthase (1e5x): LLP - coenzyme; SAM - activator. Tryptophan synthase (1bks): G3H - substrate; IDM - substrate; PLP - coenzyme; SRI - substrate. The color runs from 0 (cyan) through magenta to the maximal measured value (white).
(TIFF)

Figure S2 The leverage coupling *D_Pi_* for PFK, for one of the ADP sites, and one of the F6P sites (same as in Figure 4B and C). The filled circles indicate the residues binding either substance (in all four chains).
(EPS)

Figure S3 The leverage coupling *D_Pi_* for GTPCHI with and without GFRP, as in figure Figure 5C, D and E, but also including an analysis of one of the regulatory phenylalanine binding sites (third panel).
(EPS)

Figure S4 The leverage coupling *D_Pi_* for glycogen phosphorylase, analyzed for the active site (PLP), the regulatory AMP site, and the two sites P1 and P2 (described in main text).
(EPS)

Figure S5 The leverage coupling *D_PQ_* for thrombin.
(TIFF)

Figure S6 Leverage coupling analysis of the thermosome, similar to Figure 7 and Figure 8 for the other chaperones. The coloring of the protein surfaces indicates *D_Pi_* for one of the ATP sites, using the same scheme as in Figure 4.
(TIFF)

Figure S7 Leverage coupling *D_Pi_* for the three chaperones, analyzed for one of the 14 or 16 ATP sites in each protein.
(EPS)
