# Supplementary figures and images for: Coherent Conformational Degrees of Freedom as a Structural Basis for Allosteric Communication
Source: PLoS Comput Biol. 2011 Dec 8;7(12):e1002301. doi: 10.1371/journal.pcbi.1002301 (PMC3234217; doi:10.1371/journal.pcbi.1002301)

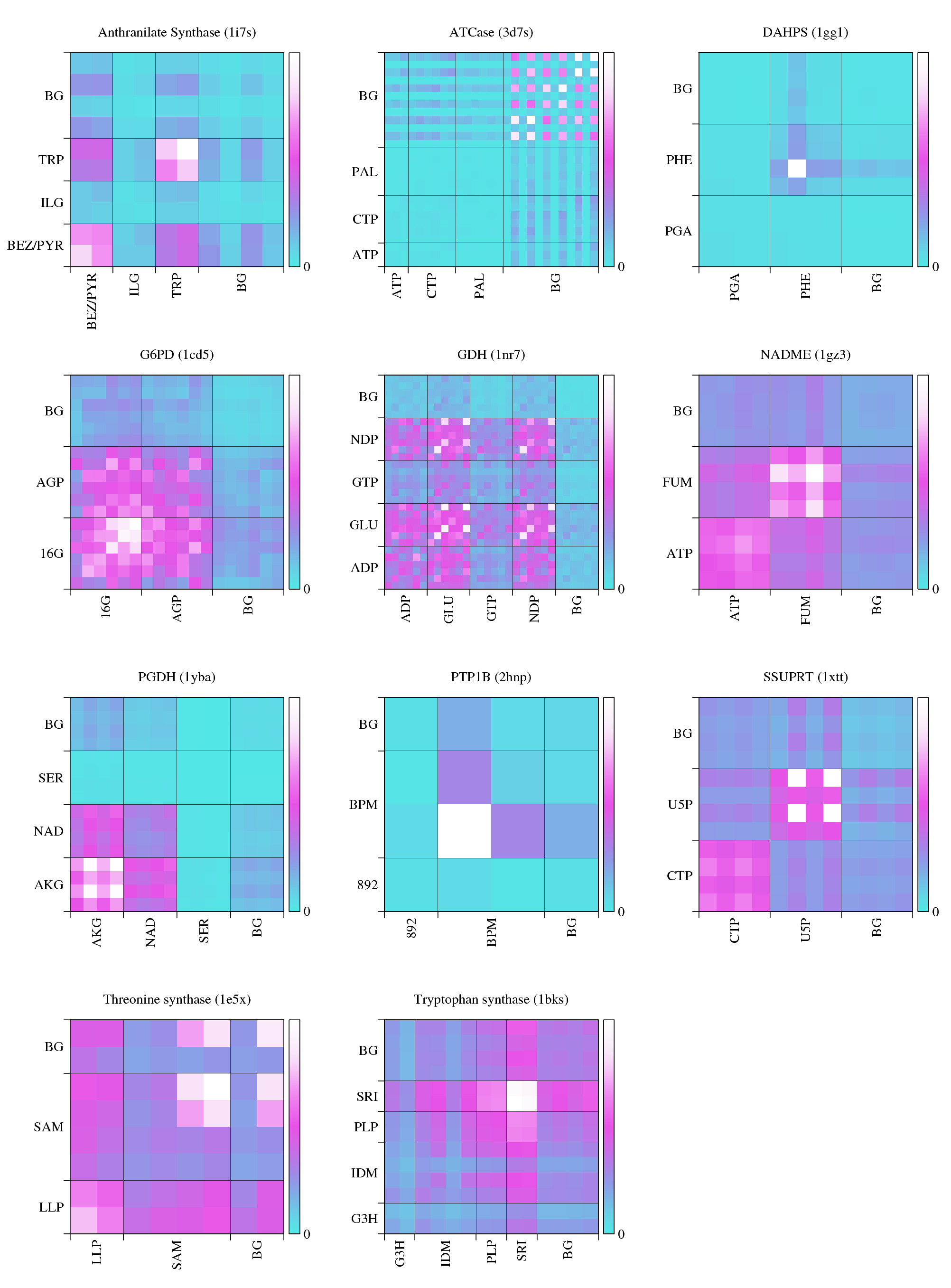

Supplement: Figure S1 — Leverage coupling DPQ for the proteins not described in the main text. The data is based on our work on binding leverage, where simulation parameters for the respective proteins can be found [15]. The label “BG” corresponds to the background, which is an average leverage coupling calculated over all residues not belonging to any site. The other abbreviations designate allosteric and functional sites, using the three letter codes found in the PDB-files for ligands binding at those sites. Anthranilate synthase (1i7s): BEZ/PYR - substrate; ILG - substrate; TRP - inhibitor. ATCase (3d7s): ATP and CTP - effectors; PAL - substrate. DAHPS (1gg1): PGA - substrate; PHE - inhibitor. G6PD (1cd5): 16G - activator; AGP - substrate. GDH (1nr7): ADP - activator; GLU - substrate; GTP - inhibitor; NDP - coenzyme. NADME (1gz3): ATP – active site ligand; FUM - activator. PGDH (1yba): AKG - substrate; NAD - coenzyme; SER - inhibitor. PTP1B (2hnp): 892 - inhibitor; BPM - substrate. SSUPRT (1xtt): CTP - inhibitor; U5P - substrate. Threonine synthase (1e5x): LLP - coenzyme; SAM - activator. Tryptophan synthase (1bks): G3H - substrate; IDM - substrate; PLP - coenzyme; SRI - substrate. The color runs from 0 (cyan) through magenta to the maximal measured value (white). (TIFF) [file pcbi.1002301.s001.tiff]

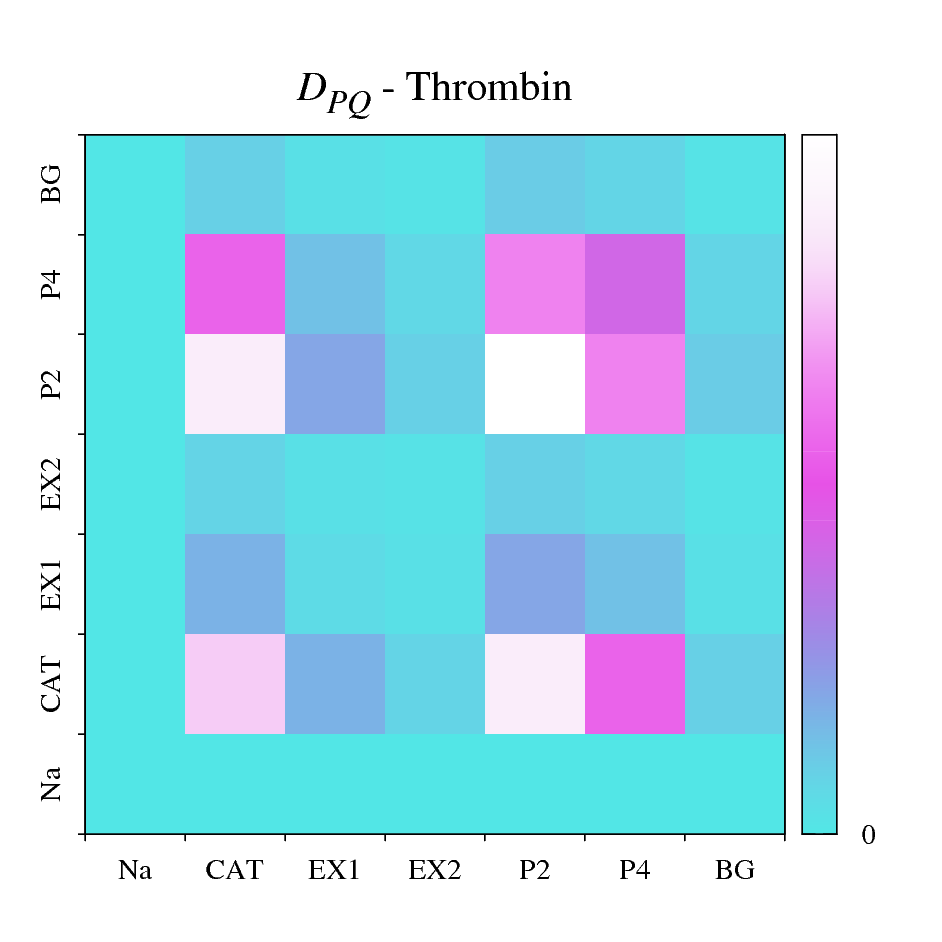

Supplement: Figure S5 — The leverage coupling DPQ for thrombin. (TIFF) [file pcbi.1002301.s005.tiff]

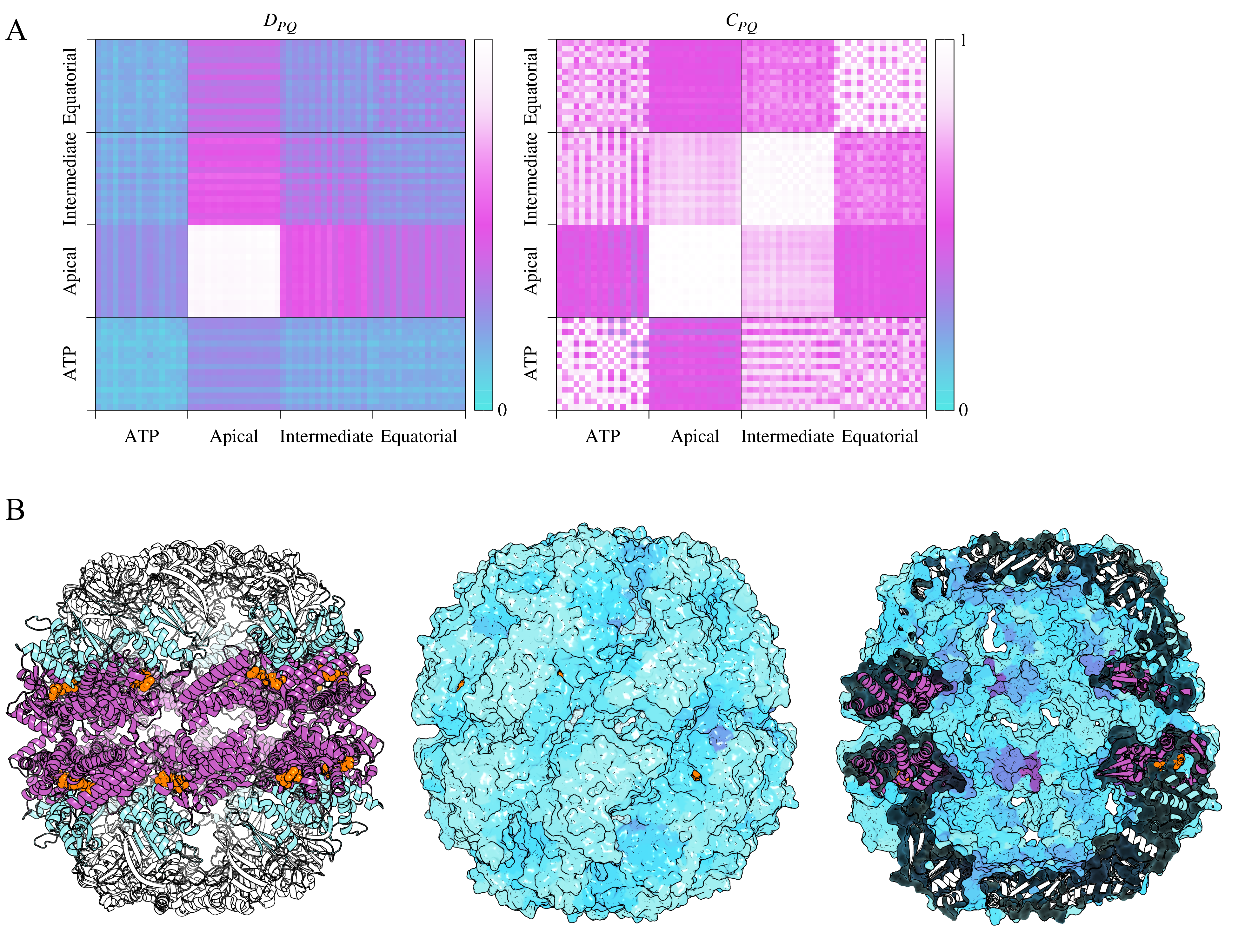

Supplement: Figure S6 — Leverage coupling analysis of the thermosome, similar to Figure 7 and Figure 8 for the other chaperones. The coloring of the protein surfaces indicates DPi for one of the ATP sites, using the same scheme as in Figure 4. (TIFF) [file pcbi.1002301.s006.tiff]
